# Do Online Privacy Concerns Predict Selfie Behavior among Adolescents, Young Adults and Adults?

**DOI:** 10.3389/fpsyg.2017.00815

**Published:** 2017-05-23

**Authors:** Amandeep Dhir, Torbjørn Torsheim, Ståle Pallesen, Cecilie S. Andreassen

**Affiliations:** ^1^Department of Teacher Education, University of HelsinkiHelsinki, Finland; ^2^Optentia Research Focus Area, North-West UniversityPotchefstroom, South Africa; ^3^Department of Psychosocial Science, University of BergenBergen, Norway; ^4^Department of Clinical Psychology, University of BergenBergen, Norway

**Keywords:** age, gender, privacy, social media, self-presentation and selfie behavior

## Abstract

Selfies, or self-portraits, are often taken and shared on social media for online self-presentation reasons, which are considered essential for the psychosocial development and well-being of people in today’s culture. Despite the growing popularity and widespread sharing of selfies in the online space, little is known about how privacy concerns moderate selfie behavior. In addition to this, it is also not known whether privacy concerns across age and gender groups influence selfie behavior. To address this timely issue, a survey assessing common selfie behaviors, that is, frequency of taking (individual and group selfies), editing (cropping and filtering), and posting selfies online, and social media privacy concerns (over personal data being accessed and misused by third parties) was conducted. The web-survey was administered to 3,763 Norwegian social media users, ranging from 13 to 50 years, with a preponderance of women (*n* = 2,509, 66.7%). The present study investigated the impact of privacy concerns on selfie behaviors across gender and age groups (adolescent, young adult, and adult) by use of the structural equation modeling approach. The results suggest that young adults have greater privacy concerns compared to adolescents and adults. Females have greater privacy concerns than males. Greater privacy concerns among female social media users were linked to lower engagement in selfie behavior, but privacy concerns did not influence selfie behavior in the case of male adolescents and young adults. Overall, privacy concerns were more consistently and inversely related to selfie behavior (taking and posting) among females than males. The study results have theoretical as well as practical implications for both researchers and policy makers.

## Introduction

People turn to online social media for various reasons including communication and self-expression, connecting, observing others, and establishing new and strengthening existing relationships (Dhir and Tsai, 2017; Dhir et al., 2017a,b). Most important of all, people use online social media to present themselves in the computer-mediated space (see Haferkamp and Krämer, 2010). Online self-presentation refers to the process of sharing content (e.g., photos, status updates, videos, and web-links) with the motive of influencing the impression formed by the people around the individual (Haferkamp and Krämer, 2010). Psychologists have argued that self-presentation is important for an individual’s well-being since it addresses their fundamental need to continuously obtain positive reactions and leave positive impressions on others (Goffman, 1959; Leary and Kowalski, 1990; Leary, 1995).

Digital photos are popularly utilized to practice online self-presentation on social media platforms (Dhir, 2016a,b). This is clearly evident from the ever-increasing growth in the number of photos shared on online social media. For example, every day nearly two billion Facebook photos alone are shared (Facebook Newsroom, 2015). Although selfies may be taken and shared in computer-mediated spaces for a number of reasons, e.g., obtaining feedback, experimentation with surrounding, etc. (Kiprin, 2013), very often selfies are shared for self-presentation reasons (Katz and Crocker, 2015). We define a selfie as a photo taken of oneself without the assistance of anyone else. Recent studies suggest that selfies have received global prominence in a short time frame (Kiprin, 2013; Katz and Crocker, 2015). The popularity of selfie sharing can be gauged from the fact that on social media, millions of selfies are shared on a daily basis. According to Weiser (2015), over 238 million photos had the hashtag #selfie, and 128 million photos had the hashtag #me on Instagram. Similarly, Svelander and Wiberg (2015) mentioned that 193 million Instagram photos and videos contain the #selfie and #selfies hashtags. This statistical evidence suggests that selfie sharing is now a dominant activity on different online social media platforms. The possible reasons behind this increase in selfie sharing on social media include identification and self-presentation (Katz and Crocker, 2015), experimentation with one’s surroundings (Kiprin, 2013), and obtaining feedback from friends, family, and peers (Katz and Crocker, 2015).

The concepts of self-presentation and self-disclosure in online social media are highly relevant, and are also strongly interrelated with each other (Haferkamp and Krämer, 2010). The precondition of online self-presentation is, to some extent, to self-disclose private information online (Boyd, 2008; Haferkamp and Krämer, 2010). However, scholars have observed a phenomenon called the “privacy paradox” which is a discrepancy between self-disclosure and privacy concerns in the computer-mediated space (Barnes, 2006). On one hand, people tend to present themselves in online space by sharing their interests, likes, tastes, hobbies, places they visit, physical appearance, etc. But on the other hand, they are wary of the potential social privacy threats (e.g., unintended exposure to a hostile or unknown audience, theft and misuse of photos) and have some degree of “privacy concern” (Livingstone, 2008). In addition to this privacy paradox, complex tensions between privacy issues and online self-presentation in a computer-mediated space also exist (Dhir, 2016a). For example, van Dijck (2008) observed that when private photos are shared in the computer-mediated space, they easily turn into public property. This rapid transformation of content from private to public space forming the desire to self-present actually results in tensions between the tendency to self-disclose and privacy concerns that are complex in nature. Similar observations were made by Boyd (2008) who found that when social media users self-disclose more personal information, it sometimes also disturbs their self-presentation choices because of social privacy issues. The different social privacy threats to personal online self-presentation goals include exposure to unknown people, negative criticism from peers, and being tagged in an unattractive, inaccurate, undesirable, and/or low quality self-presentation (Lang and Barton, 2015; Dhir et al., 2016a). Scholars have also emphasized that such social privacy disturbances in online self-presentation goals also result in online regret (i.e., negative cognitive experience) (Wang et al., 2011; Kaur et al., 2016a), and negative feedback on perceived social and self-identity (Lang and Barton, 2015). It can even affect continuous service use and customer retention (Dhir, 2016a). These studies have found that privacy concerns and privacy issues significantly influence users’ online self-presentation choices and decisions. However, it is currently unknown to what extent “privacy concerns” predict specific online self-presentation behavior or acts.

According to the privacy paradox phenomenon, selfie sharing also involves some degree of self-disclosure of current activities, emotions, hobbies, and interests. At the same time, however, it makes those people wary of their actions, and they also have some degree of privacy concern. Furthermore, when private selfies are shared in the computer-mediated space, they turn into public property and generate tensions, which are complex in nature, between privacy concerns, self-presentation goals and social privacy threats. Despite the fact that “privacy concerns” can possibly affect user experience and different choices pertaining to online self-presentation, surprisingly little is known about the relationship between self-presentation and online privacy concerns. Furthermore, selfie sharing in the computer-mediated space is becoming more and more popular; yet, it is not known how privacy concerns influence selfie-related behavior. It is important to understand this relationship because it informs the researchers and practitioners of how privacy concerns predict online self-presentation-related behavior, which is considered a dominant activity in online social media. Furthermore, better understanding of this relationship can potentially provide new insights into the complex relationship between online privacy concerns and self-disclosure (i.e., the privacy paradox) in computer-mediated systems (see Boyd, 2008; Madden and Smith, 2010; McLaughlin and Vitak, 2012). This study has addressed this open research gap through the investigation of the relationship between privacy concerns and selfie-related behavior as a means of online self-presentation. To date, scholars have investigated different issues pertaining to online self-presentation and privacy, but the novelty of the current study lies in its investigation of the relationship between privacy concerns and user behavior pertaining to online self-presentation, which has not as yet been studied. Hence, the current study contributes to the emerging literature on selfie-related behavior as well as the extant literature on online self-presentation and privacy.

The prior social media literature has been criticized due to its overemphasis on United States (US) based study participants (Dhir et al., 2015; Dhir, 2016a; Kaur et al., 2016b). However, the vast majority of social media users are actually based outside of the US; for example, over 84.2% of Facebook users are in fact based outside of the US (Facebook Newsroom, 2015). Another limitation of the previous literature is the emphasis on the social media behavior of young-adult samples only (Dhir, 2016a). However, significant age differences in social media usage patterns have been reported. For example, adolescents differ from young and older adults in their use of social media (Andreassen et al., 2016; Dhir and Torsheim, 2016). Similarly, these groups also differ in terms of selfie-posting (Sorokowski et al., 2015; Dhir et al., 2016b) and online privacy behavior (Madden and Smith, 2010; Xie and Kang, 2015). Due to these two inherent shortcomings, only limited understanding of social media behavior (particularly selfie-related and online privacy) across age and cultures is available. Furthermore, only a few studies have set out to investigate the differences in the social media behavior of different age groups; for example, adolescents vs. young adults vs. older adults. Consequently, the transferability of the prior study findings across a broad age range is not known.

The current study has addressed these research gaps by examining the impact of privacy concerns on selfie behavior in the context of three target user groups: adolescent, young-adult, and adult social media users. This study also addresses the pressing need to investigate the selfie behavior of mixed age/gender groups (Albury, 2015) since the overwhelming majority of the prior literature has focused on single gender samples only (Nelson, 2013; Nguyen, 2014; Warfield, 2014). The main research questions of the current study are: **RQ1**. How do adolescents, young adults, and adults differ in their privacy concerns regarding social media? **RQ2**. How do males and females differ in their privacy concerns regarding social media? **RQ3**. How do privacy concerns across genders (male and female) and age groups (adolescent, young adult, and adult) predict selfie behavior (taking, editing, and posting selfies)?

## Background Literature

### Online Privacy Concerns and Age Differences

The prior computer-mediated communication literature suggests significant age differences in privacy behavior. Young-adult social media users possess high levels of privacy concern, and they tend to disclose less information than both older and younger (e.g., adolescent) users (Nosko et al., 2010). The study by Madden and Smith (2010) found that young-adult social media users (18–29 years) have more experience of managing privacy of online shared content, and are more likely to use privacy-preserving strategies (e.g., changing default privacy settings, limiting access to the shared content, and cropping photos to hide personal information) than older (50–64 years) social media users (41% vs. 18%). This is also consistent with the observations of Strano and Wattai (2010) who found that privacy-preserving strategies (e.g., untagging) are more popular among young adults (aged 18–21 years), compared to older (above 31 years) social media users (66.4% vs. 14.5%). Similarly, Dhir et al. (2016a) claimed that older adolescents are more likely to untag compared to their younger counterparts. Lang and Barton (2015) found that young adults actively engage in management of their social privacy. In comparison to young adults, adolescents are known to self-disclose themselves more often on social media compared to when they are offline (Schouten et al., 2007). Feng and Xie (2014) also suggested that adolescent social media users have lower social privacy concerns and therefore have greater willingness to self-disclose online compared to young adults. Several studies have indicated that, compared to young adults, adolescents tend to disclose more personal information and do so more frequently (Livingstone et al., 2010; Xie and Kang, 2015). Similarly, Madden et al. (2013) observed that adolescents with high privacy concerns actually post more content online. The possible reasons could be that adolescents lack up-to-date understanding of the different privacy-related settings (Christofides et al., 2012), they may face technical glitches when managing their online content (Brandtzæg et al., 2010), or there may be differences in the use of computer-mediated technologies among younger and older users (Hayes et al., 2015). However, despite all of these studies, it is not known at present how these three user groups, adolescents, young adults, and adults, differ in their privacy concerns regarding social media. Therefore, we propose the following hypotheses based on the prior literature:

H1: Young-adult social media users possess greater privacy concerns than adult users.
H2: Adult social media users possess greater privacy concerns than adolescent users.

### Online Privacy Concerns and Gender Differences

Similar to age differences, several studies have suggested significant gender differences in privacy behavior. Young-adult men are known to self-disclose relatively more personal information online since they do not foresee associated social privacy concerns (Fogel and Nehmd, 2009), and are likely to experience online regret due to high self-disclosure (Moore and McElroy, 2012) compared to female young-adult users. Similar observations have been made in the case of adolescents, as males disclose more personal information online than female adolescents (Xie and Kang, 2015). In terms of privacy-preserving strategies, Dhir et al. (2016a) found that female adolescents are less likely to use privacy-preserving strategies (e.g., untagging) than male adolescents. This is contrary to the case of young adults, as female young adults have been found to be more likely to use privacy-preserving strategies compared to male young adults (McLaughlin and Vitak, 2012; Tufekci, 2012). However, how this compares with the gender differences in privacy behavior among adults (aged 30–50 years) is presently unknown. There is still a lack of understanding of how males and females across the three age groups (adolescent, young adult, and adult) differ in their privacy concerns regarding social media. Based on the limited available literature, we propose the following hypotheses:

H3: Female adolescents possess greater privacy concerns than male adolescents.
H4: Female young adults possess greater privacy concerns than male young adults.
H5: Female adults possess greater privacy concerns than male adults.

### Age and Gender Differences in Selfie Behavior

Selfies are popular among adolescents (Senft and Baym, 2015) as well as among young adults (Katz and Crocker, 2015). Using a convenience sample, Katz and Crocker (2015) found that 96% of 20- to 23-year-old young adults had taken selfies in the recent past, while 25% had taken a selfie only the day before. Furthermore, 98% of 18- to 24-year-olds had taken selfies, 46% had shared selfies only the day before, while 69% of the young people shared selfies 3–20 times per day. Similarly, Dhir et al. (2016b) found that adolescents were the most active and adults were the least active social media users in taking and posting selfies. These studies articulate that taking and sharing selfies are very popular pastimes and are part of the daily routine of adolescents and young adults. However, how this compares with the popularity of selfies among adults (aged 30–50 years) is presently unknown. A handful of recent studies have suggested significant age differences in selfie behavior. The older population is less likely to take selfies compared to their younger counterparts (Qiu et al., 2015) due to less of a desire to fulfill their narcissistic objectives (Weiser, 2015). Furthermore, young adults are less concerned as to how posting selfies on social media will affect them in the future compared to older adults (Katz and Crocker, 2015). Several studies have indicated a positive relationship between online self-presentation and selfie taking and posting in the context of adolescent social media users (Senft and Baym, 2015).

Selfies are taken and shared for the identification of gender and self-presentation reasons (Katz and Crocker, 2015), and several studies have suggested significant gender differences in selfie behavior. Albury (2015) also emphasized that selfie taking and posting behavior is a gendered process in which females tend to receive unfair criticism and are inappropriately targeted and scrutinized due to their provocative selfie posting (Albury, 2015). Similarly, Burns (2014) argued that females are typically viewed as objects of consumption, while males are not subjected to such surveillance or scrutiny. On this issue, Williams and Marquez (2015) argued that due to gender role stereotypes (e.g., Rudman and Glick, 2001), people who tend to violate the gender code (i.e., Anderson, 1999) actually receive negative feedback from their peers.

Scholars have found that selfies are relatively more popular among females, and that they are more likely to take selfies than males (Qiu et al., 2015). To begin with, Sorokowski et al. (2015) found that females post more personal and group selfies compared to men. Albury (2015) also found the existence of gender differences in the linguistic aspects of any selfie post. Poe (2015) noticed that high self-esteem was associated with posting of more selfies among young-adult women, while Cao and Halloran (2014) observed that, compared to men, selfies taken by women were more personal in nature. Young women (18- to 29-year-olds) tend to share selfies to obtain positive feedback (Nguyen, 2014). Nelson (2013) found that young women use the hashtags #me, #selfie, and #self in their selfies for self-presentation reasons (e.g., obtaining positive feedback). Furthermore, young women tend to worry after sharing selfies if they fail to attract sufficient positive feedback (e.g., number of likes) (Nelson, 2013). In a recent study, Dhir et al. (2016b) found that female social media users are more likely to take personal and group selfies and to post personal selfies. Similarly, Warfield (2014) found that “policing” selfie-taking and sharing are popular among younger women (aged 16–28 years). In comparison to these findings, Fox and Rooney (2015) observed that men who are self-objectifying are more likely to spend time on social media and to frequently edit photos. Furthermore, men who are relatively more narcissistic and psychopathic are more likely to share edited photos and selfies and to engage in impulsive posting of selfies on social media, which tends to attract the attention of their peers. This review of the prior literature clearly suggests the importance of age and gender in selfie behavior.

### Online Privacy Concerns and Self-Presentation

The prior findings on the empirical linkages between online privacy concerns and self-disclosure are not consistent, as some studies have indicated that there is no significant relationship between them (Stutzman and Kramer-Duffield, 2010; Forest and Wood, 2012). However, in comparison, a recent study by Xie and Kang (2015) observed that social media users with high levels of privacy concern actually disclose more personal information, consistent with the ‘privacy paradox’ phenomenon. The relationship between privacy concerns and activities pertaining to online self-presentation is also unclear. For example, people with high privacy concerns do not necessarily maintain a low self-presentation profile, but usually conceal part of the content shared online (e.g., cropping or hiding) (Haferkamp and Krämer, 2010). Therefore, it is currently unknown how privacy concerns predict different user activities pertaining to online self-presentation. Similarly, it is also not known how gender and age differences impact the influence of privacy concerns on the online self-presentation behavior of the three different age groups, that is, adolescent, young-adult, and adult social media users. These issues are addressed in the present study. Based on the prior literature on “age and gender differences” in online privacy (see “Background Literature”), we propose the following hypotheses:

H6: Privacy concerns do not play any significant role in influencing the selfie behavior of male adolescents.
H7: Privacy concerns play a significant role in influencing the selfie behavior of male young adults.
H8: Privacy concerns play a significant role in influencing the selfie behavior of male adults.
H9: Privacy concerns do not play any significant role in influencing the selfie behavior of female adolescents.
H10: Privacy concerns play a significant role in influencing the selfie behavior of female young adults.
H11: Privacy concerns play a significant role in influencing the selfie behavior of female adults.

## Materials and Methods

### Study Participants and Procedure

This current study is based on a large-scale national cross-sectional web-survey, which explored several on-/offline behaviors of the general population in Norway. The survey was broadcast (July-August 2015, for a period of 1 week) by two nationwide news media delivering news to the general public (i.e., a television news broadcast and an online newspaper).

The survey featured and focused on the use and overuse of online social media (with no specific reference to selfie behavior or privacy concerns regarding social media). Respondents could enter the survey by clicking on an open-access web-link providing access to the survey. The first page provided the complete details of the study, that is, study objectives and process, ethical considerations, and anticipated outcomes. In order to participate, the respondents were required to confirm by actively entering “yes” (the other option was “no” which led to a page just thanking them for their interest). At the end of the survey, immediate feedback on scores was used to design engaging obtainer experiences, to which each participant was given informative content of general and specific interest. There were no other incentives for participation. Consent to participate was considered as “given” if a participant successfully completed the questionnaire. The responses obtained from the participants were stored in the database of the Internet survey company and were later passed over to our researchers. In conducting the study, we followed the Norwegian Health Research Act and the ethical guidelines of the Helsinki Declaration. According to the guidelines of the Norwegian Health Research Act, if the data collection is anonymous (voluntary and non-interventional) then approval from the Norwegian Social Science Data Service and the Regional Committee for Medical and Health Related Research Ethics is not required.

A total of 4,126 social media users (*M*_age_ = 30.45, *SD*_age_ = 13.00, range 13–82 years) participated in the study. Out of the total valid sample, 3,763 (*M*_age_ = 27.79, *SD*_age_ = 10.05) social media users represented three groups: adolescents (*M*_age_ = 16.96, *SD*_age_ = 1.74, range 13–19 years, 398 males and 570 females), young adults (*M*_age_ = 24.18, *SD*_age_ = 3.07, range 20–30 years, 478 males and 994 females), and adults (*M*_age_ = 39.73, *SD*_age_ = 5.66, range 31–50 years, 378 males and 945 females). This data-set was also used in another study for investigating the age and gender differences in selfie-related behavior (Dhir et al., 2016b).

### Study Measures

#### Selfie Behavior

Selfie behavior was assessed using five items. The first two addressed the frequency of selfie-taking: “How frequently do you take individual selfies” and “How frequently do you take group selfies.” Both of these items were taken from Sorokowski et al. (2015). The third item examined the frequency of selfie-posting: “How frequently do you post individual selfies on social media.” This item was taken from the prior selfie literature (Fox and Rooney, 2015; Weiser, 2015). The last two items assessed photo-editing behavior and were adapted from Fox and Rooney (2015): “How frequently do you post photos on social media that are cropped in order to make you look better,” and “How frequently do you post photos on social media after using photographic filters to make you look better.” These items were evaluated using a 5-point response scale ranging from 1 (*never*) to 5 (*always*).

#### Social Media Privacy Concerns

Social media privacy concerns were evaluated using four items based on the work of Dinev and Hart (2006). The items were: “I am concerned that the information I share on social media could be misused,” “I am concerned that others can find private information about me on social media,” “I am concerned about providing personal information on social media, because of what others might do with it,” and “I am concerned about sharing personal information on social media, because it could be used in a way I did not foresee.” The four items are addressed using the following descriptive statements throughout the manuscript – “Fear of information misuse,” “others find private information,” “others might use my private information,” and “use of private information is unforeseen.” The social media privacy concerns were evaluated on a 5-point response scale ranging from 1 (*completely disagree*) to 5 (*completely agree*).

### Data Analysis

**FIGURE 1:**
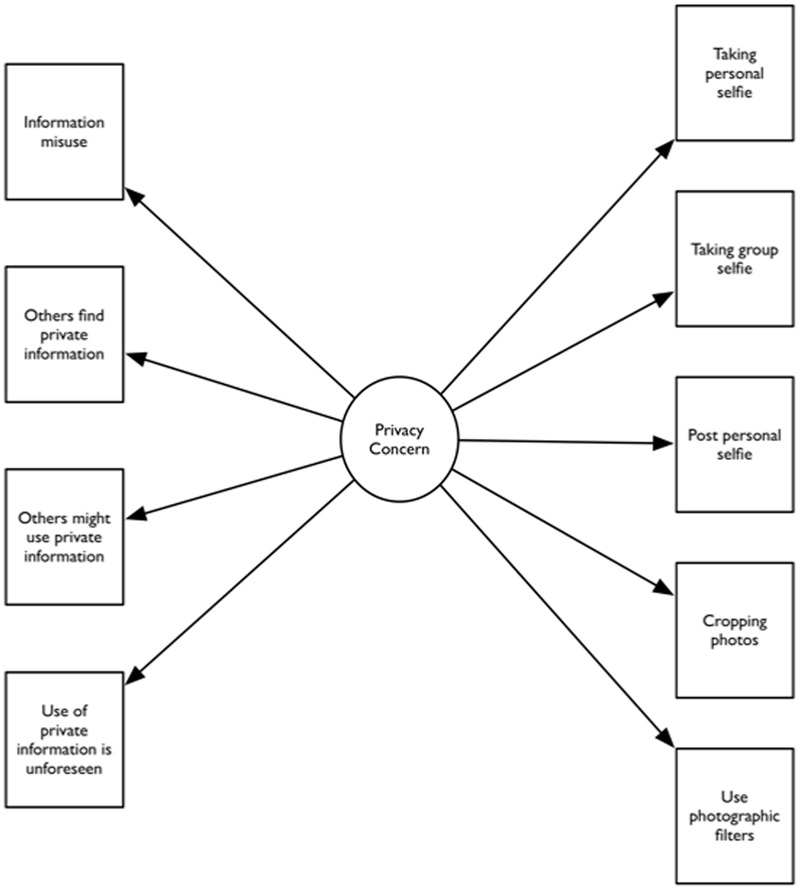
**Privacy concerns and selfie behavior model**.

The statistical software Mplus was used for the data analysis. The data did not deviate strongly from a normal distribution across the three age groups because skewness and kurtosis were in the range of ±1 (Byrne, 2001; George and Mallery, 2003). All *Z*-scores were below 3.29 (recommended threshold limit), hence the data were considered free from any potential outliers (Tabachnick and Fidell, 2007). Multigroup structural equation modeling (SEM) was performed in order to examine how privacy concerns influence different selfie behaviors across age and gender groups. It was postulated that the four items on privacy concerns reflected a common latent privacy concerns factor. According to the model, privacy concerns predicted the five dependent variables on selfie behaviors, namely individual selfie-taking, group selfie-taking, selfie-sharing frequency, photo-cropping, and use of photographic filters. The theoretical model of the study is presented in **Figure 1** (omitting error terms). In line with previous validation studies, the theoretical model was used as baseline model. To evaluate sources of model misspecification, modification indices (Sörbom, 1989) were computed. Strong modification indices would suggest a need for model revision. Prior SEM literature suggests that scholars should evaluate the goodness of model fit for the baseline model and revisions of the baseline model (Khazaal et al., 2011, 2012). This is important to address the possible discrepancy between the empirical data and the hypothetical model which tends to exist in most scenarios.

## Results

**Table 1** presents the mean and standard deviation values for selfie behavior across age and gender groups.

**Table 1:** Means (M) and Standard Deviations (SD) for selfie behavior.

Selfie behavior	*M*(*SD*)					
	Male			Female		
	Adolescents	Young adults	Adults	Adolescents	Young adults	Adults
Taking personal selfies	2.46 (1.06)	2.33 (0.92)	2.05 (0.77)	3.09 (0.99)	2.82 (0.87)	2.36 (0.82)
Taking group selfies	2.26 (0.91)	2.11 (0.88)	1.83 (0.76)	3.08 (0.90)	2.58 (0.85)	2.22 (0.77)
Posting personal selfies	1.85 (0.89)	1.77 (0.84)	1.76 (0.76)	2.49 (0.90)	2.17 (0.83)	2.01 (0.75)
Cropping photos	1.74 (1.01)	1.82 (1.07)	1.68 (0.85)	2.48 (1.12)	2.29 (1.04)	1.96 (0.91)
Using photographic filters	2.13 (1.18)	2.03 (1.08)	1.56 (0.84)	3.08 (1.14)	2.70 (1.20)	1.79 (0.94)

The baseline structural equation model (**Figure 1**) resulted in a poor model fit where χ^2^ = 1585.71, *df* = 132, χ^2^/*df* = 12.01, comparative fit index (*CFI*) = 0.92, Tucker–Lewis Index (*TLI*) = 0.86, and root mean square error of approximation (*RMSEA*) = 0.13. The recommended values for goodness of model fit are as follows: χ^2^/*df* < 3, *CFI ≥* 0.92, *TLI* ≥ 0.92, and *RMSEA* < 0.08 (Hu and Bentler, 1999; Byrne, 2001). The modification index between the error terms of the “fear of information misuse” and “others find private information” items was very high, suggesting a possible discrepancy between the empirical data and the hypothetical model. The revised model, including correlated error terms of “fear of information misuse” and “others find private information,” revealed a good model fit where χ^2^ = 199.48, *df* = 125, χ^2^/*df* = 1.60, *CFI* = 0.996, *TLI* = 0.993, and *RMSEA* = 0.03. **Table 2** presents the standardized factor loadings for the “privacy concern” items in the revised measurement model.

**Table 2:** Standardized factor loadings for privacy concern in the constrained measurement model.

Privacy concern	Standardized estimate					
	Male			Female		
	Adolescents	Young adults	Adults	Adolescents	Youngadults	Adults
Fear of information misuse	0.71	0.76	0.75	0.67	0.67	0.70
Others find private information	0.79	0.81	0.82	0.75	0.72	0.76
Others might use my private information	0.88	0.93	0.91	0.91	0.90	0.89
Use of private information is unforeseen	0.83	0.89	0.88	0.87	0.88	0.87

**FIGURE 2:**
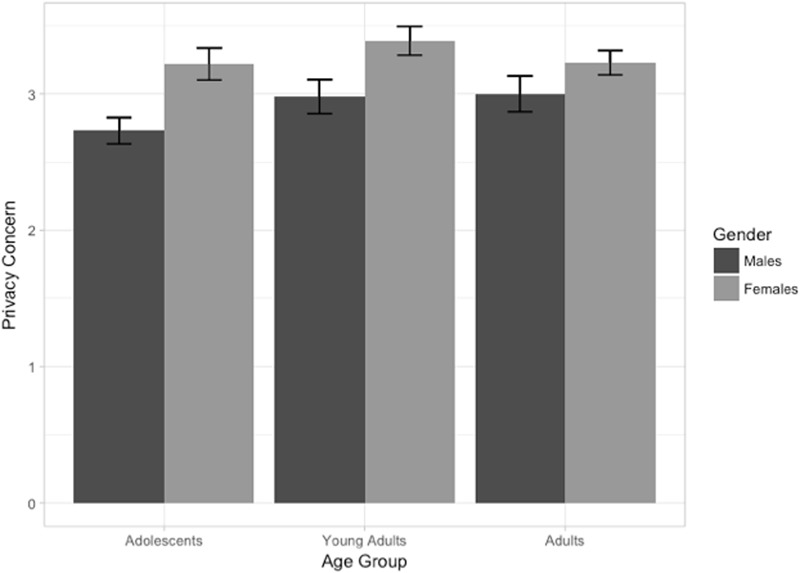
**Mean of Privacy concerns across age groups and gender.** Limits of error bars denote 95% confidence interval for the mean.

**Figure 2** presents the estimated latent mean scores of privacy concerns across age and gender groups. Young-adult social media users had greater privacy concerns in comparison to the other two age groups, and the adult social media users had greater privacy concerns than the adolescent social media users. For the gender variable, the study results suggest that females have greater privacy concerns than male social media users across all three age groups.

**Table 3** presents the regression weights for selfie behaviors as observed dependent variables regressed on latent privacy concern. The SEM results revealed that privacy concerns did not significantly predict selfie behaviors of the male adolescents and young adults. However, privacy concerns significantly predicted “taking group selfies” (*p <* 0.01) and “remaining selfie behavior” (*p <* 0.05) among male adult users. In comparison to this, privacy concerns significantly predicted taking personal selfies, cropping or editing photos, and use of photo-filters among the adolescent, young-adult and adult female social media users. However, privacy concerns significantly predicted posting personal selfies only among young-adult and adult females. Furthermore, privacy concerns significantly predicted taking group selfies only among adult female social media users (see **Table 3**).

**Table 3:** Standardized regression weights from structural equation model of selfie behaviors regressed on privacy concerns.

DV: Selfie behavior	Beta values for IV: Privacy concerns					
	Male			Female		
	Adolescents	Young adults	Adults	Adolescents	Young adults	Adults
Taking personal selfies	-0.06	-0.03	-0.11^∗^	-0.11^∗^	-0.13^∗∗^	-0.09^∗∗^
Taking group selfies	-0.10	-0.01	-0.16^∗∗^	-0.06	-0.07	-0.15^∗∗^
Posting personal selfies	-0.09	-0.01	-0.13^∗^	-0.09	-0.09^∗∗^	-0.16^∗∗^
Cropping photos	-0.02	-0.04	-0.11^∗^	-0.17^∗∗^	-0.08^∗^	-0.14^∗∗^
Using photographic filters	-0.06	-0.02	-0.11^∗^	-0.14^∗∗^	-0.10^∗∗^	-0.08^∗^

*^∗^*p* < 0.05 and ^∗∗^*p* < 0.01; IV, independent variable; DV, dependent variable.*

## Discussion

The present study investigated the role of online privacy concerns in influencing selfie behavior of social media users across gender and age groups, that is, adolescent, young-adult, and adult social media users. A large self-selected sample of online social media users based in Norway was recruited in order to investigate the three main research questions of the current study. The novelty of this work lies in the focus on two important yet less studied variables: age-gender differences in selfie behavior in the computer-mediated space. The present study further examined the complex, obscure, and rarely studied relationship between privacy concerns and online self-presentation (i.e., selfie behavior in this study). Such investigation is timely as well as being much needed since it addresses the urgent demand to understand the differences in selfie behavior across gender and broader age groups [see the recent work by Sorokowski et al. (2015)]. Furthermore, the current study also addresses the long-standing demand of prior computer-mediated literature to examine social media behavior among culturally and geographically diverse groups of users (Dhir et al., 2015; Kaur et al., 2016b). The study results suggest that (a) young adults have greater privacy concerns compared to both adults and adolescents, (b) greater privacy concerns among females were not linked to lower engagement in selfie behavior, and (c) privacy concerns among male adolescents and young adults did not influence their selfie behavior. In addition to this, the study findings indicate that privacy concerns were more consistent and inversely related to the selfie behavior of female social media users than of their male counterparts.

The first research question (**RQ1**) investigated the differences in the perceptions of privacy concerns among adolescent, young-adult, and adult social media users. The results suggest that young-adult (20–30 years of age) social media users have greater privacy concerns compared to the other age groups, and that adult social media users are much more concerned about their privacy compared to adolescent users. Therefore, both hypotheses H1 and H2 were supported. These findings are consistent with the findings reported in the prior literature. For example, scholars have observed that young-adult social media users are more experienced in managing their online privacy (Madden and Smith, 2010; Young and Quan-Haase, 2013; Lang and Barton, 2015), and in utilizing privacy-preserving strategies (Strano and Wattai, 2010), as well as having a greater level of privacy concerns (Nosko et al., 2010) than older adults. Similarly, the prior literature also suggested that adolescent social media users possess lower online privacy concerns (Feng and Xie, 2014), self-disclose themselves online more often (Schouten et al., 2007), post content more frequently (Livingstone et al., 2010; Xie and Kang, 2015), and post more online content despite their privacy concerns (Madden et al., 2013; Feng and Xie, 2014) than young adults. These findings also suggest that “age differences in privacy concerns” are similar to age differences in the management of online privacy and related issues. Possible explanations for these findings include: First, adolescents do not possess sufficient understanding of “privacy concerns” (Christofides et al., 2012), and face technical problems in understanding and later in the translation of their privacy concerns into practice (Brandtzæg et al., 2010). Second, young adults have a better understanding of online privacy-related issues due to which they have greater “privacy concerns.”

The second research question (**RQ2**) examined the differences in the privacy concerns of male and female social media users. The study results suggest that female users have greater privacy concerns than male users across all three age groups. Therefore, hypotheses H3, H4, and H5 were supported. These findings are consistent with the prior literature, in that male young adults tend to self-disclose more and to have relatively lower privacy concerns (Fogel and Nehmd, 2009; Moore and McElroy, 2012) compared to female young adults. Similarly, male adolescents are known to self-disclose more personal information online compared to their female counterparts (Xie and Kang, 2015). These findings suggest that: (1) female social media users have greater privacy concerns across all three age groups, and (2) The use of privacy management strategies has no influence on the degree of privacy concerns among male and female social media users. For example, the prior literature suggests that male young adults are less likely (Hoy and Milne, 2010; McLaughlin and Vitak, 2012; Tufekci, 2012), and male adolescents are more likely to engage in privacy-preserving strategies than their respective female counterparts (Dhir et al., 2016a).

The third research question (**RQ3**) investigated how privacy concerns across the three age groups (adolescents, young adults, and adults) and the two gender groups (male and female) influence selfie behavior (i.e., selfie-taking, sharing, and photo-editing).

The study results indicate that for male adolescents and male young adults, privacy concerns play an insignificant role in influencing selfie behavior. However, in the case of adult social media users, greater privacy concerns translate into lower engagement in selfie-taking and sharing frequency, and in photo-editing behavior. Therefore, H6 and H8 were supported but H7 was not supported. The study finding that privacy concerns among male adolescents and young adults do not influence their selfie behavior is consistent with the prior literature, which has also indicated that male social media users self-disclose much more and have relatively lower privacy concerns (Fogel and Nehmd, 2009; Moore and McElroy, 2012; Xie and Kang, 2015). The study findings also indicate that although male adults have relatively greater privacy concerns than adolescents and lower privacy concerns than young adults, the privacy concerns of adults tend to moderate their selfie behavior because their greater concerns lead to lower engagement in selfie behavior. These findings suggest that: (1) greater privacy concerns do not necessarily translate into lower or higher engagement in selfie-related behavior (i.e., online self-presentation), and (2) adult social media users were the least active of the three age groups in terms of selfie behavior, and had lower privacy concerns than young adults, but their privacy concerns still translated into lower engagement in selfie-related behavior.

In the case of female social media users, the results suggest that greater privacy concerns result in lower engagement in taking personal selfies, cropping photos (i.e., a privacy-preserving strategy) and use of photographic filters among all three age groups, including adolescent, young-adult, and adult female social media users. Therefore, H10 and H11 were supported, but H9 did not receive support from the results. This is consistent with the findings of the prior literature which indicated that female social media users are relatively active in terms of monitoring their privacy settings (Hoy and Milne, 2010), and engage in privacy-preserving strategies (Pempek et al., 2009; Strano and Wattai, 2010). However, the present study results extend this understanding because it indicates that privacy concerns moderate the selfie-related behavior (i.e., online self-presentation) of female social media users, where greater privacy concerns result in lower engagement in taking personal selfies, cropping/editing photos, and use of photo-enhancement filters.

In terms of group selfie-taking, the study results suggest that privacy concerns among female adolescent and young-adult social media users were a non-significant predictor, unlike the case of female adult users. A possible explanation for this could be that female adolescents and young-adult social media users actively engage in group selfie-taking as a means of showcasing as well as strengthening peer membership because it is part of their self-identity development, and is considered very important to them (see Senft and Baym, 2015). Due to their urge to engage in peer membership via group selfies, privacy concerns do not influence their group selfie behavior.

Regarding personal selfie posting behavior, the study results suggest that privacy concerns were statistically non-significant in the case of female adolescents, but were a statistically significant predictor in the case of female young-adult and adult social media users. One possible reason could be that adolescent females tend to post personal selfies as a means of experimenting with self-identity and self-presentation (Gibbs et al., 2014) due to which they are not bothered about their privacy concerns. Therefore, privacy concerns among female adolescents do not influence their personal selfie posting behavior.

Comparing the two gender groups across the three age groups, the results for the adolescent social media users suggest that privacy concerns did not influence group selfie taking or personal selfie posting behavior among either male or female users. This suggests that group selfie taking and personal selfie posting are part of the online self-presentation behavior that is considered very important for the well-being and development of adolescents (Dhir, 2016b). Due to their desire to self-present themselves in the computer-mediated space, privacy concerns do not influence their online self-presentation choices. In terms of taking personal selfies, cropping photos, and using photographic filters, the results suggest that privacy concerns were a significant predictor among female but not male adolescents. This suggests that privacy concerns among female adolescents are a significant predictor of privacy-preserving strategies (e.g., cropping of photos) and online self-presentation (i.e., taking personal selfies), which is consistent with the prior literature (see McLaughlin and Vitak, 2012; Tufekci, 2012).

In the case of young-adult social media users, privacy concerns did not predict group selfie-taking among either male or female young adults. As mentioned before, one possible reason could be that group selfies are associated with peer membership, which is important for young adults. Due to this reason, young adults are less bothered about privacy concerns when it comes to taking group selfies. In comparison, privacy concerns did not influence taking and posting selfies, cropping photos, or using photographic filters among male young adults, whereas they did for female young adults. This is again consistent with the findings of the prior literature, which suggests that females are more concerned about online privacy than male social media users (Pempek et al., 2009; Hoy and Milne, 2010).

Finally, in the case of adult social media users, the results suggest that privacy concerns resulted in lower engagement in selfie-taking and selfie-sharing frequency and in photo-editing behavior across both male and female users. The possible reasons could be that older adults are more concerned that selfie taking and posting will affect them negatively in the future (Katz and Crocker, 2015).

### Study Implications

The present study has different theoretical and practical implications for both scholars and practitioners. In terms of its theoretical implications, the current study findings contribute significantly to the interdisciplinary literature on human–computer interaction, new media, computer-mediated communication, as well as developmental psychology. Second, the present findings complement the available qualitative findings (e.g., Nelson, 2013; Nguyen, 2014; Warfield, 2014) on the age and gender differences in selfie behavior with quantitative results. Similarly, the study results also contribute to the sporadic literature on age and gender differences in the computer-mediated communication space. Third, the study findings provide new understandings of the social media use and selfie behavior of the lesser-studied cultural group of Norwegian social media users. Therefore, it contributes to broadening the limited literature on cross-cultural studies on social media use behavior. Fourth, this study has addressed the long-standing need to investigate the social media use behavior (including selfie behavior) of mixed age and gender groups because the prior literature focused on young-adult social media users only. Fifth, the study findings provide deeper insights into the age and gender differences in privacy concerns among social media users. Furthermore, the study results provide crucial insights into how privacy concerns influence online self-presentation choices, that is, selfie behavior in the present study. Sixth, the present study concludes with insightful findings on the influence of privacy concerns across gender (male and female) and age groups (adolescent, young adult, and adult) in predicting the selfie-related behavior of social media users. The practical implications of the current study include: New insights and knowledge for the various stakeholders such as social media and service companies, startups, online service and mobile application designers, developmental psychologists and researchers, and developers who are interested in capitalizing on the popularity of selfie behavior for business gains. Second, the study results can help these different stakeholders to understand the importance of age and gender differences among social media users, which will enable them to tap age- and gender-specific user markets with ease. Consequently, the study findings will enable them to understand their existing as well as prospective customers. Third, the study findings may motivate scholars to investigate the age and gender differences in the important issues concerning the computer-mediated communication space, including user-generated content, online communities, and massive online sharing and tagging. Similar investigations will bring more clarity to and understanding of the obscure and complex age and gender differences existing in the computer-mediated space. Fourth, the present study findings could possibly offer innovative space and also initiate discussion for redefining the discourse on social, personal, legal and policy-making discussions regarding online self-presentation (consistent with Albury, 2015) and the relevant age and gender related differences in the use of computer-mediated platforms.

### Study Limitations and Future Work

The main limitation of the current study is the self-selected nature of the sample, which was recruited via two leading online news media. Due to this, it is likely that two user groups, namely young adults and adults, were over-represented. This is mainly because online newspapers are particularly popular among young adults and adult Internet users. However, the sample sizes across the three groups were comparable. Of note, the two news entities are nationwide rather than local news media, which increases the possibility of reaching out to a broad range of Norwegian people. Norwegians are also known for being heavy newsreaders, as well as having broad access to the Internet. Nevertheless, we still recommend that other scholars validate the study findings using more representative study samples. Any future investigation with similar research questions should also try to generalize the findings to other age groups, particularly adults older than 50 years. In addition to this, other possible future directions include expanding the study focus to other aspects of selfie behavior such as selfies classified as public, private, and romantic (coined by Sorokowski et al., 2015). Similarly, other aspects of selfie behavior related to online self-presentation should be investigated in future studies. One example is studies on how social media users attribute personality traits to other users based on photos and other displayed social network profile characteristics (Mazza et al., 2015), and also to which extent the users’ self-reported personality matches the attributed traits. We would also like to emphasize the dynamic nature of social media. Therefore, future investigations should investigate the longitudinal effect on the user patterns in selfie and social media behavior.

## Conclusion

Overall, the present study offers important insights into the relationship between privacy and self-presentation, including selfie behavior in the computer-mediated communication space. It further highlights the importance of studying age and gender differences in selfie behavior. In addition to this, the study complements and extends the available findings in the context of age and gender differences in computer-mediated environments, privacy concerns, and selfie behavior among adolescent, young-adult, and adult social media users.

## Author Contributions

SP, CA, and AD led the conception and design of the study. CA led the data collection. AD led the literature search, analysis, interpretation of the data, drafting, writing, and revising the work. All authors contributed to the design (AD, CA, SP, and TT), analysis (AD, SP, and TT), interpretation of data (AD, CA, SP, and TT), and/or writing and revising the work critically for important intellectual content (AD, CA, SP, and TT). All authors read and approved the final version of the work to be published (AD, CA, SP, and TT), and agreed to be accountable for all aspects of the work in ensuring that questions to the accuracy of any part of the work are appropriately investigated and resolved (AD, CA, SP, and TT).

## Conflict of Interest Statement

The authors declare that the research was conducted in the absence of any commercial or financial relationships that could be construed as a potential conflict of interest.
